# Gait Dynamics in Parkinson’s Disease: Short Gait Trials “Stitched” Together Provide Different Fractal Fluctuations Compared to Longer Trials

**DOI:** 10.3389/fphys.2018.00861

**Published:** 2018-07-09

**Authors:** Vivien Marmelat, Nicholas R. Reynolds, Amy Hellman

**Affiliations:** ^1^Center for Research in Human Movement Variability, Department of Biomechanics, University of Nebraska Omaha, Omaha, NE, United States; ^2^Department of Neurological Sciences, University of Nebraska Medical Center, Omaha, NE, United States

**Keywords:** walking, gait variability, Parkinson’s disease, detrended fluctuation analysis, scaling exponent, nonlinear dynamics, gait analysis

## Abstract

The fractal analysis of stride-to-stride fluctuations in walking has become an integral part of human gait research. Fractal analysis of stride time intervals can provide insights into locomotor function and dysfunction, but its application requires a large number of strides, which can be difficult to collect from people with movement disorders such as Parkinson’s disease. It has recently been suggested that “stitching” together short gait trials to create a longer time series could be a solution. The objective of this study was to determine if scaling exponents from “stitched” stride time series were similar to those from continuous, longer stride time series. Fifteen young adults, fourteen older adults, and thirteen people with Parkinson’s disease walked around an indoor track in three blocks: one time 15 min, five times 3 min, and thirty times 30 s. Stride time intervals were determined from gait events recorded with instrumented insoles, and the detrended fluctuation analysis was applied to each stride time series of 512 strides. There was no statistically significant difference between scaling exponents in the three blocks, but intra-class correlation revealed very low between-blocks reliability of scaling exponents. This result challenges the premise that the stitching procedure could provide reliable information about gait dynamics, as it suggests that fractal analysis of stitched time series does not capture the same dynamics as gait recorded continuously. The stitching procedure cannot be considered as a valid alternative to the collection of continuous, long trials. Further studies are recommended to determine if the application of fractal analysis is limited by its own methodological considerations (i.e., long time series), or if other solutions exists to obtain reliable scaling exponents in populations with movement disorders.

## Introduction

Consecutive gait cycles during steady-state walking are not exactly identical. Slight changes from one step to the next create stride-to-stride fluctuations over time, i.e., gait variability. Gait variability refers not only to the magnitude of fluctuations, assessed by measures of dispersion around the central tendency (e.g., standard deviation or CV), but also to the serial correlations between consecutive strides (i.e., *temporal ordering* of fluctuations) (Pierrynowski et al., 2005; Damouras et al., 2010; Dingwell and Cusumano, 2010). Serial correlations can be assessed using the detrended fluctuation analysis (DFA; Peng et al., 1993), which is often preferred to other methods due to its robustness to non-stationarities and its greater accuracy for relatively short time series, e.g., a few hundred cycles (Chen et al., 2002; Delignieres et al., 2006; Almurad and Delignières, 2016; Kuznetsov and Rhea, 2017). Briefly, the DFA assesses the relationship between the average magnitude *F* of a time series fluctuations and the window *n* over which these fluctuations are computed. When the relationship obeys the power-law *F(n) ∼ n*^α^, the fluctuations present scale invariant fractal properties (i.e., fluctuations are statistically similar over different time scales), and a linear fit in log-log coordinates reveals the scaling exponent α. The value of the DFA scaling exponent α indicates the nature of serial correlations: for stationary time series, α > 0.5 indicates statistical persistence (i.e., large fluctuations are likely to be followed by larger fluctuations, and vice-versa), α < 0.5 indicates anti-persistence (i.e., large fluctuations are likely to be followed by smaller fluctuations, and vice-versa), and α = 0.5 indicates the absence of serial correlations (i.e., random process similar to white noise).

The presence of serial correlations in spatiotemporal gait parameters (e.g., stride time intervals) may reveal significant insights into neuromuscular control of locomotion (Goldberger, 1997; Hausdorff et al., 1997, 2000, 2001; Hu et al., 2004; Baltadjieva et al., 2006; Stergiou and Decker, 2011; Roerdink et al., 2015). Stride-to-stride fluctuations in HY adults present persistent fluctuations characterized by a single scaling exponent α, i.e., monofractal fluctuations (Hausdorff et al., 1995, 1996; Terrier et al., 2005; Delignières and Torre, 2009; Marmelat et al., 2014). This suggests that locomotor control is not achieved on a “step-by-step” basis, but rather on a “multi-scale” basis (Ashkenazy et al., 2002; Goldberger, 2006; Stergiou et al., 2006; Ivanov et al., 2009; van Orden et al., 2009; Delignières and Marmelat, 2012), where each stride is dependent on previous (and statistically speaking, future) dozens or even hundreds of strides. While the origins of serial correlations in stride time series of healthy human gait are unclear, several models support the hypothesis that they may emerge from non-linear interactions between multiple networks acting at different time scales (Hausdorff et al., 2001; Ashkenazy et al., 2002; Ivanov et al., 2009; Delignières and Marmelat, 2013).

With aging, the scaling exponent α tends to decrease toward randomness (Goldberger, 1996; Hausdorff et al., 1997; Hausdorff, 2007), indicating less correlated strides, as if each gait cycle was the result of a new process. Notably, these findings are consistent with the hypothesis that the reduction of serial correlations in stride-to-stride fluctuations may originate from reduced connectivity and activation of certain neural networks (Hausdorff et al., 2001; Ashkenazy et al., 2002; Ivanov et al., 2009; Delignières and Marmelat, 2013). Lower scaling exponents have also been associated with greater risk of falls in the elderly (Hausdorff, 2007), suggesting a close relationship between serial correlations and adaptability (Goldberger et al., 2002; Harbourne and Stergiou, 2009; van Orden et al., 2009). Importantly, changes in scaling exponent are independent from changes in average stride time and in the magnitude of fluctuations. Neurological dysfunctions affecting neural rhythm generation also alter the temporal ordering of stride-to-stride fluctuations (Hausdorff et al., 1997; Hove et al., 2012; Uchitomi et al., 2013; Ota et al., 2014; Moon et al., 2016; Warlop et al., 2016, 2018). In particular, patients with PD exhibit less persistent fluctuations in stride time series compared to age-matched controls, suggesting greater impairments in the regulation of gait timing. Warlop et al. (2016) recently evidenced a negative correlation between PD severity and the scaling exponent from stride time series. They also reported a positive correlation between PD severity and the CV of stride time series, suggesting that PD gradually impairs locomotor stability. These results confirm that the scaling exponent α provides crucial information about the locomotor system’s integrity.

A major limitation in the application of DFA is the time series length (Chen et al., 2002; Delignieres et al., 2006; Kuznetsov and Rhea, 2017). A large number of gait cycles are required to obtain a reliable estimation of serial correlations in stride-to-stride fluctuations. Delignieres et al. (2006) applied DFA to simulated time series and highlighted that time series shorter than 512 values would increase the variability of the scaling exponent estimates beyond acceptable levels. Empirical results lead to similar conclusions: for example, Damouras et al. (2010) proposed to use at least 600 stride intervals (i.e., around 10 min walking) to accurately differentiate normal and pathological walking. This requirement can become problematic for people with PD who may not be able to safely walk unassisted for such a long time. In fact, with the exception of Warlop et al. (2016), the majority of studies investigating serial correlations in PD populations collected between 2 and 5 min of data during a single trial (Hausdorff et al., 2000; Baltadjieva et al., 2006; Hove et al., 2012; Uchitomi et al., 2013; Moon et al., 2016; Dotov et al., 2017), which may be too short to draw relevant conclusions about the impact of PD on the temporal ordering of stride-to-stride fluctuations.

Different solutions have been proposed to address the limitation of the application of DFA to “short” behavioral time series. Pierrynowski et al. (2005) proposed to average the scaling exponents obtained from multiple shorter gait trials (e.g., three 6-min or two 8-min trials). However, the accuracy of each estimate using this method could lead to averaging together unreliable scaling exponents and potentially misleading conclusions. Another technique has been proposed to overcome the problem of time series length, namely the “stitching” procedure (Kirchner et al., 2014), which consists in combining consecutive short gait trials to create a longer time series, potentially more suitable for the application of DFA. Many authors warned that estimating *serial* correlations requires continuous recording, i.e., *serial* collection of data, and that the stitching procedure would create artificial changes in the scaling exponent α (Chen et al., 2002; Pierrynowski et al., 2005; Marmelat et al., 2012; Kuznetsov and Rhea, 2017). However, the majority of studies investigating serial correlations in PD gait were conducted in hallways, where subjects walked back and forth, and a median filter was applied afterward to remove outliers corresponding to the turning points (Hausdorff et al., 2000; Baltadjieva et al., 2006; Hove et al., 2012; Uchitomi et al., 2013). This procedure is conceptually similar to stitching each portion of the gait trial between turning points. The application of this procedure was probably motivated by findings from Chen et al. (2002) who evidenced that removing up to 50% of (simulated) signals with persistent fluctuations did not significantly impact the scaling exponent. In line with these findings, and under certain conditions (e.g., collecting short trials consecutively, within the same experimental environment), the stitching procedure may provide relevant information about neuromuscular control of locomotion in populations not able to walk for a long time, such as patients with PD.

While the stitching procedure may appear promising to analyze serial correlations in stride time series from clinical populations unable to walk continuously for a long time, the results from Kirchner et al. (2014) present many limitations—that the authors themselves acknowledged—to confidently apply the stitching procedure in clinical settings. In particular, in their study, (i) the “stitched” time series contained only 125 strides, which is probably too short for a proper estimation of the scaling exponent α; (ii) the “stitched” time series were not compared to longer time series recorded continuously; (iii) only one “short trial” duration was tested (i.e., 25 strides), but it is unknown if the length of the trial would influence the final outcome of α values; (iv) PD patients were compared to HY adults, leaving a potential confounding effect of age. The present study aims to address these limitations in order to identify if the stitching procedure could provide relevant information about gait variability in people with PD, healthy older adults, and healthy younger adults.

Based on previous literature, we make the assumption that PD patients will present lower DFA values under every experimental condition compared to HY and healthy older adults. In addition, based on the results from Kirchner et al. (2014), we make the hypothesis that the stitching procedure will tend to slightly increase DFA values. Finally, we predict that the stitching procedure will disrupt the serial correlations in individual stride time series, resulting in low between-conditions reliability of DFA values. We will also compare the experimental results to artificially stitched time series based on the continuously recorded time series, to evaluate the effects of the “start and stop” walking on DFA values.

## Materials and Methods

### Ethics

This study was approved prior to its conductance by the Institutional Review Board of the University of Nebraska Medical Center. This study was carried out in accordance with the recommendations of the Institutional Review Board at the University of Nebraska Medical Center with written informed consent from all subjects. All subjects gave written informed consent in accordance with the Declaration of Helsinki.

### Participants

After giving written informed consent, 15 elderly patients with PD, 15 HE adults, and 15 HY adults participated in the experiment. One participant in the HE group and two participants in the PD group were excluded from further analyses for different reasons (cf. Results section). Therefore, analyses were applied to 15 HY, 14 HE, and 13 PD. To qualify in the study, all subjects needed to be able to ambulate independently without external support for at least 20 min. Subjects in the PD group and the HE group needed to be at least 50 years old, while subjects in the HY group needed to be between 19 and 35 years of age. To qualify in the PD group, subjects must have been diagnosed with idiopathic PD as defined by the UK Brain Bank Criteria. Subjects with known orthopedic disease, legal blindness, lower extremity vascular disease, cardiac disease, pulmonary disorders, known neurological disease (other than PD for the PD group), depression (evaluated using the Geriatric Depression Scale; Yesavage et al., 1983), mild cognitive dysfunction (evaluated using the Montreal Cognitive Assessment; Nasreddine et al., 2005), balance impairment (evaluated using the Fullerton Advanced Balance scale, Rose et al., 2006; the Modified Falls Efficacy Scale, Yardley et al., 2005; and the Timed Up-and-Go test, Podsiadlo and Richardson, 1991), and subjects who had experienced a fall in the last year (determined by self-report) were excluded. PD patients with deep-brain stimulation, known neurological disease other than PD or freezing of gait (evaluated using the Freezing of Gait Questionnaire; Giladi et al., 2000) were also excluded. Subjects in the PD group were assessed under their regular medication (“on-state”). Subject demographics and results from clinical and functional tests are displayed in **Table 1**.

**Table 1:** Demographic and clinical characteristics of participants.

	HY (*N* = 15)	HE (*N* = 14)	PD (*N* = 13)
Number of men/women	10/5	5/9	11/2
Age (years)	23.00 ± 1.69	67.71 ± 5.92	71.31 ± 6.12
Height (cm)	177.67 ± 8.21	166.68 ± 8.33	169.75 ± 10.01
Weight (kg)	78.51 ± 13.42	72.58 ± 13.01	76.45 ± 14.16
Fullerton Advanced Balance scale	39.87 ± 0.52	38.00 ± 2.14	35.71 ± 4.10
Timed Up and Go (s)	5.74 ± 1.55	7.48 ± 1.14	9.07 ± 2.66
Montreal Cognitive Assessment	28.53 ± 1.73	27.87 ± 2.00	26.00 ± 3.33
Geriatric Depression Scale	–	0.47 ± 0.74	1.50 ± 1.87
Freezing of Gait score	–	–	4.71 ± 4.14
Hoehn and Yahr scale	–	–	1.64 ± 0.69

*Mean ± standard deviations for: HY, healthy young; HE, healthy elderly; PD, patients with Parkinson’s disease. Welch’s *t*-test did not reveal any statistically significant differences between HE and PD groups for any variables.*

### Apparatus and Equipment

Participants walked in all conditions with four force-sensitive footswitches placed directly under the heel and the forefoot of each foot, in order to collect initial contact (heel-strike) and final contact (toe-off) of each stride taken. The sample rate for each footswitch was set at 1942 Hz. Subjects were instructed to wear comfortable clothing and comfortable shoes.

### Tasks and Procedure

The study consisted of 45 min total of walking, divided into three blocks performed in a randomized order. The three blocks consisted of i) one 15-min trial (“15 min” block), ii) five 3-min trials (“3 min” block), and iii) thirty 30-s trials (“30 s” block). Consequently, participants walked 15 min within each block. At least 5 min of rest was provided between blocks, and at least 30 s of rest was provided between trials in the “3 min” and “30 s” blocks. Subjects were instructed to walk at a regular, self-selected speed around a 200-m long indoor track in all trials. They received no other instructions nor any feedback regarding their walking speed between trials or between blocks. During the “3 min” and “30 s” blocks, the procedure was as follow: the experimenter instructed participants to start walking along the track, and started recording. When the trial ended (i.e., after 30 s or 3 min), the experimenter stopped recording and instructed participants to stop. Participants waited for 30 s before starting another trial along the track in the same direction, as instructed by the experimenter. Participants remained standing during the 30 s resting period between trials. They had the opportunity to sit on a chair during the 5 min resting period between blocks. During all trials, an experimenter walked behind participants, within a distance that did not interfere with participants’ gait but close enough to be ready to intervene in case of loss of balance. There was no visual evidence of freezing of gait nor festinating gait in any of subjects with PD.

### Data Processing

**FIGURE 1:**
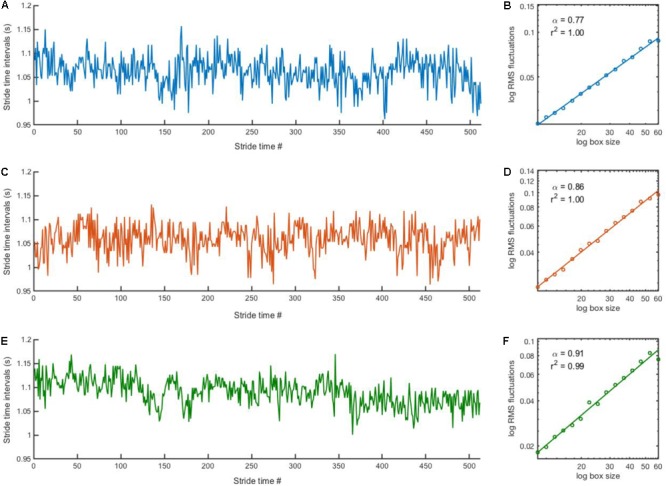
Stride time series and corresponding DFA plot. Representative examples of stride time series (left) and corresponding DFA plot (right) from a participant in the PD group in the “15 min” **(A,B)**, “3 min” **(C,D)** and “30 s” **(E,F)** blocks. Note that it is impossible to distinguish where the stitching occurred for the “3 min” **(C)** and “30 s” **(E)** time series.

The main dependent variable in this study was the scaling exponent of the series of stride-time intervals. A stride time interval was defined as the time between a gait event (heel-strike or toe-off) of a foot to the time of the next same gait event of that same foot. Stride times were identified using a customized code created in Matlab©R2017a, and a stride time interval was simply defined as the difference between two consecutive stride times. Five strides were removed at the beginning and the end of each individual trial to reduce the effects of gait initiation and gait deceleration. In order to create longer stride time series, the five trials in the “3 min” block and the 30 trials in the “30 s” block were, respectively, “stitched” together. For example, if one 30 s trial consisted of about 32 strides, stitching 30 trials of 22 strides (i.e., 32 minus the first and last five strides) resulted in a longer series that would contain 660 stride time intervals (i.e., 22 × 30 = 660). The stitching procedure simply consisted in concatenating consecutive trials via a customized Matlab©code. In order to reliably compare data from all participants in all blocks, only the first *N* stride time intervals from the longer time series were analyzed, where *N* is the length of the shortest time series across all participants in all blocks. This resulted in comparing time series of 512 stride time intervals (**Figure 1**, left panels; Supplementary Data S1), which should be long enough to obtain reliable DFA results (Delignieres et al., 2006).

The scaling exponent α of the series of stride-time intervals was estimated using the detrended fluctuation analysis (DFA; Peng et al., 1993). DFA was selected because it provides good results for “short” time series (Chen et al., 2002; Delignieres et al., 2006; Kuznetsov and Rhea, 2017). The methodology of DFA has been described in detail elsewhere (Peng et al., 1993; Almurad and Delignières, 2016). Briefly, the signal is first integrated after subtracting the global average. The time series is then divided into non-overlapping boxes of length *n*. Within each box of size *n*_i_, a least-square polynomial fit of order one is applied to the integrated signal to remove the local trend. The root-mean-square fluctuation function F(*n*_i_) quantifies the fluctuations of the integrated signal along the local trend, yielding the average magnitude of fluctuation F(*n*). This process is repeated across all size of *n*, ranging from 10 to *N*/8, where *N* is the time series length. A linear relationship between F(*n*) and *n* in log-log coordinates (**Figure 1**, right panels) characterizes the presence of a power-law in the time series, i.e., scale invariance. The slope of this linear relationship determines the scaling exponent α, as *F(n) ∼ n*^α^, which quantifies the degree of persistence in the signal. If α = 0.5, there is no correlation between successive values and the signal is uncorrelated (i.e., white noise). If α < 0.5, the fluctuations are anti-persistent (i.e., negative correlation between successive values) and if α > 0.5 the fluctuations are persistent (i.e., positive correlation between successive values).

### Statistics

A two-way ANOVA (3 groups × 3 blocks) was used to compare the following measures from stride time intervals: mean, CV, and α-DFA. *Post hoc* analysis entailed Tukey’s multiple comparison’s tests. For each group, ICC coefficient (ICC 2,1) was performed to determine the reliability of mean, CV, and α-DFA between the three blocks. The reliability was graded with ICC values ranging below 0.40 indicating poor reliability, values from 0.40 to 0.59 indicating fair reliability, values from 0.60 to 0.74 indicating good reliability, and values from 0.75 to 1.00 indicating excellent reliability (Cicchetti, 1994). Level of statistical significance was set at a *p*-value < 0.05.

### Artificially Stitched Time Series

Stride time series from the “15 min” block (512 stride intervals) were first divided into four segments of 128 strides or 32 segments of 16 strides (to mimic the “3 min” and “30 s” trials, respectively). The order of these segments was then randomized for each participant, and stitched back together to re-create a longer time series. We then compared the ICC of DFA values from the original “15 min” trials, the time series resulting from four randomized segments (“rand_4”) and the time series resulting from 32 randomized segments (“rand_32”). This process was repeated 20 times for each group, resulting in 20 ICC values that were averaged for each group.

## Results

One subject in the HE group was excluded from further analyses because he did not complete the 5th “3 min” trial. Two subjects in the PD group were also excluded because footswitches did not record properly the subject’s heel strikes. For the remaining 15 HY, 14 HE, and 13 PD subjects, results from right and left stride time series were not significantly different (*p* > 0.05); therefore, only results from right stride time series are reported.

### Effect of Groups and Blocks on Measures

#### Mean

The two-way ANOVA revealed no interaction [*F*(4,78) = 0.6225, *p* = 0.6478] nor blocks [*F*(2,78) = 0.1152, *p* = 0.8913] effects. A significant group effect was detected [*F*(2,39) = 5.131, *p* = 0.0105]. *Post hoc* analysis revealed that stride time means were significantly lower in the HE group compared to the HY group (mean difference = 0.084 s, *p* = 0.008).

#### CV

The two-way ANOVA revealed no interaction [*F*(4,78) = 0.2918, *p* = 0.8825] nor blocks [*F*(2,78) = 0.663, *p* = 0.5182] effects. A significant group effect was detected [*F*(2,39) = 3.817, *p* = 0.0306]. Although *post hoc* analyses did not detect any statistically significant differences, stride time CV were greater in the PD group compared to the HE group (mean difference = 0.4518 %, *p* = 0.0543) and to the HY group (mean difference = 0.4498 %, *p* = 0.0509).

#### DFA

**FIGURE 2:**
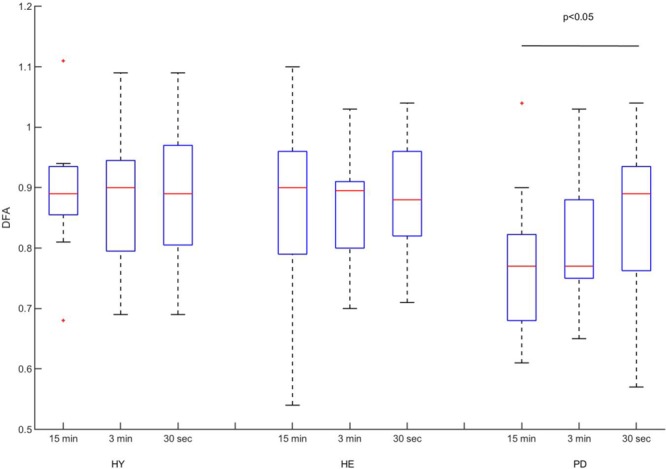
Experimental DFA values. Classical boxplots were used to indicate median, quartiles, and extent of the data (whiskers). Red crosses represent outliers. Results from the two-way ANOVA indicated a significant main effect of group (*p*-value reported in the figure). Mean values and standard deviation of mean, coefficient of variation, and DFA can be found in **Table 2**.

The two-way ANOVA revealed no interaction [*F*(4,78) = 0.5429, *p* = 0.7047] nor blocks [*F*(2,78) = 0.8418, *p* = 4348] effects. A significant group effect was detected [*F*(2,39) = 3.628, *p* = 0.0359]. *Post hoc* analysis revealed that stride time DFA values were significantly lower in the PD group compared to the HY group (**Figure 2**; mean difference = 0.08, *p* = 0.0496). A non-significant difference between HE and PD groups was also observed (mean difference = 0.07, *p* = 0.0745).

### Between-Block Reliability of Measures

**FIGURE 3:**
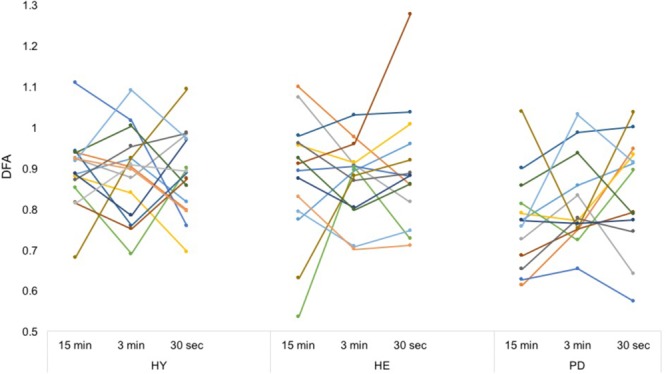
Individual DFA values. Each color represents a participant. From a simple visual inspection, it is apparent that while mean values are similar, each participant’s DFA values differ a lot between conditions, without any consistent trend. Intra-class correlation coefficients can be found in **Table 2**.

Between-blocks consistency of the mean and CV of stride time series was excellent for all groups (**Table 2**), with the exception of the CV in the HE group. In contrast, between-blocks consistency of the DFA was poor to fair for all groups (**Figure 3**).

**Table 2:** Mean (SD) of stride time series mean, standard deviation and DFA values, and corresponding intra-class correlation coefficients (95% confidence interval) for each group.

		15 min	3 min	30 s	ICC (95% CI)
HY	Mean (s)	1.066 (0.071)	1.067 (0.070)	1.068 (0.066)	0.95 (0.90–0.98)
	CV (%)	1.76 (0.46)	1.73 (0.53)	1.84 (0.44)	0.83 (0.65–0.93)
	DFA	0.89 (0.09)	0.89 (0.11)	0.88 (0.11)	–0.07 (–0.28 to 0.28)
HE	Mean (s)^∗^	0.985 (0.064)	0.984 (0.068)	0.978 (0.072)	0.93 (0.85–0.98)
	CV (%)	1.71 (0.49)	1.83 (0.56)	1.79 (0.39)	0.29 (–0.03 to 0.65)
	DFA	0.87 (0.16)	0.87 (0.09)	0.90 (0.15)	0.33 (0.00–0.67)
PD	Mean (s)	1.016 (0.080)	1.009 (0.082)	1.014 (0.083)	0.96 (0.90–0.99)
	CV (%)	2.20 (0.85)	2.20 (0.53)	2.29 (0.69)	0.74 (0.48–0.90)
	DFA^∗^	0.77 (0.12)	0.81 (0.11)	0.84 (0.14)	0.43 (0.08–0.74)

*^∗^Statistical significant difference compared to HY (*p* < 0.05).*

### Artificially Stitched Time Series

**FIGURE 4:**
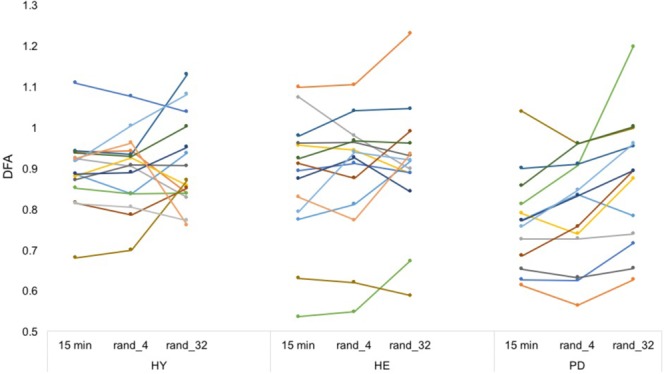
Artificially stitched individual DFA values. Each color represents a participant. From a simple visual inspection, DFA values seem rather reliable between conditions (in contrast to **Figure 3**). Note that the procedure was repeated 20 times to enhance the estimations. Intra-class correlation coefficients can be found in Supplementary Material S2.

Averaged ICC values for HY, HE, and PD groups were equal to 0.64 (±0.076), 0.86 (±0.038), and 0.81 (±0.031), respectively (**Figure 4** and Supplementary Data S2).

## Discussion

The aim of this study was to determine if stitching together short gait trials to create a longer stride time series would provide similar serial correlations compared to continuously recorded stride time series. Our major finding is that there was no significant effect of the experimental conditions on any of the variables, suggesting that gait variability measures—in particular DFA values—were not affected by the stitching procedure *on average*. However, a more careful inspection using ICC coefficients revealed that DFA values were not consistent between blocks in any group. This suggests that the stitching procedure alters *individual* serial correlations and therefore should not be considered as a simple alternative to longer continuous gait trials.

Detrended fluctuation analysis values were slightly lower in the PD group compared to the HE group, but this difference was not statistically significant (*p* = 0.0745). While other studies have reported lower DFA values for stride time series in people with PD (Hausdorff et al., 2000; Baltadjieva et al., 2006; Hove et al., 2012; Uchitomi et al., 2013; Moon et al., 2016), different factors may have played a role to explain the absence of statistical significant differences between our HE and PD groups. For example, the ability to walk for at least 20 min unassisted—suggesting low levels of gait impairment for participants in the PD group—was an eligibility criteria for the study. In addition, subjects in the PD group presented on average a Hoehn and Yahr score of 1.64 (**Table 1**), typical of the early stage of the disease (Hoehn and Yahr, 1967). Ota et al. (2014) suggested that stride time CV was primarily affected by PD in the early stages, and that impairments in serial correlations could be a more sensitive marker of disease progression in later stages. Accordingly, in this study the PD group exhibited (non-statistically significant) greater CV of stride time series (>2%) compared to the HE group (<2%). Impairments in CV and DFA seem related to increased risk of falls (Hausdorff, 2007; Warlop et al., 2016). However, both the origins of these deficits and their respective role for gait stability and adaptability remain to be determined. We advocate that more frequent longitudinal assessment of gait variability measures will provide a better understanding of PD progression and its impact on gait stability and risk of falls (Baltadjieva et al., 2006; Weiss et al., 2014; Warlop et al., 2016).

Contrary to our hypothesis, the stitching procedure did not increase DFA values. Kirchner et al. (2014) suggested that the procedure would create a pseudo-structure which in turn would increase the persistent trend in the time series. While DFA values in the PD group seemed to slightly increase as the length of short bout trials decreased (**Table 2**), this difference was not significant. The absence of statistically significant differences between blocks could—at first glance—suggest that the DFA provides similar information about locomotor control when time series are recorded continuously or created from shorter gait trials stitched together.

However, ICC coefficients revealed only poor to fair between-blocks reliability of DFA values (**Table 2**). This result is particularly relevant in the context of gait rehabilitation. The stitching procedure has been proposed as an alternative to longer gait trials, which may not always be possible for different reasons (e.g., population unable to walk for a long time, or experimenters having access only to short hallways). However, our results do not support this proposition. DFA values collected continuously in the “15 min” block can be considered as the reference to which other DFA values should be compared. It must be stressed that 15 min is considered the reference in the present study, not as an absolute “gold-standard” time series length. Theoretically, scale invariance implies that the scaling exponent from different length of the same time series should be similar, but empirical evidences in the context of locomotion are missing. Nonetheless, 15 min seems a good compromise to meet the requirements of DFA without inducing other factors such as increased fatigue or declined attention. ICC coefficients clearly evidenced that individual DFA values were not consistent between blocks. As such, DFA results from stitching procedures should not be considered as alternate versions of the continuous, longer gait trial. It is important to note that if DFA values were consistently over- or underestimated with the stitching procedure *for all* subjects, experimenters could take this consistent bias into account. However, our results did not show such a consistent trend. For each group, DFA values tended to remain within the same range between conditions, but each individual produced either greater, lower, or similar DFA values between conditions, with no consistent trend.

The results from the artificially stitched time series (i.e., time series from the “15 min” block divided in smaller segments and stitched back in random order) provide complementary information to explain our experimental results. For the three groups, ICC values were good to excellent, in contrast to the experimental ICCs (**Table 2**). This suggests that during the experimental conditions, starting and stopping during each 3 min or 30 s trials lead to slightly different gait dynamics. This is consistent with previous literature (Kuznetsov and Rhea, 2017) suggesting that DFA values resulting from the stitching procedure are difficult to interpret, because the serial correlations between strides “truly” exists only at the scale of a trial (i.e., 15 min, 3 min, or 30 s). Conversely, one may argue that serial correlations may exist between events produced by the same (locomotor) system under similar constraints, even if the activity is interrupted. In theory, the same—or at least very similar—neural networks are responsible for gait rhythmicity (Lo et al., 2017), and it is plausible that long-range correlations exist between non-continuous dynamics (e.g., walking, stopping, and crossing a street). While our results suggest that DFA values need to be applied from a continuously recorded time series, further studies may investigate the temporal long-term correlations between non-continuous dynamics.

Very few studies examined the between-trial reliability of DFA under similar conditions. This validation is necessary before comparing DFA values obtained from different experimental conditions. While data collected during over-ground walking are missing, both within-day and between-day ICC coefficients were excellent (0.914 and 0.769, respectively) for time series recorded during 8-min treadmill walking, i.e., roughly 400 strides (Pierrynowski et al., 2005). Recently, Choi et al. (2015) reported a high within-day and an excellent between-day reliability of stride time DFA recorded for 10-min on a feedback-controlled treadmill. These results suggest that stride time DFA values remain stable for a given individual during treadmill walking, and tend to support our claim that the stitching procedure itself critically alters DFA values. Further studies testing the reliability of stride time DFA over-ground will be necessary before our conclusions can be entirely validated.

This study presents several limitations. First, participants in this study walked over-ground, but as mentioned previously, previous studies investigating the reliability of stride time DFA values focused on treadmill-walking. In addition, while over-ground walking is ecologically relevant, treadmill walking is important in both research and clinical settings, and can potentially improve gait in people with PD (Frenkel-Toledo et al., 2005; Earhart and Williams, 2012; Mirelman et al., 2016; Warlop et al., 2018). Further studies should therefore investigate the potential of the stitching procedure during treadmill walking. Another limitation is related to demographics: all subjects in our PD group were in the early stages of the disease, and able to walk unassisted for at least 20 min. This precludes the generalization of our results to more advanced stages of the disease, or to other movement disorders. However, the stitching procedure did not provide reliable scaling exponents even for the unaffected populations (HY and HE groups), suggesting that the lack of reliability is independent of subjects’ health condition. We also did not control for potential confounding effects of Parkinson’s medications (e.g., Levodopa) on gait (Lord et al., 2011). Levodopa is known to increase step length and reduce CV in stride time and stride length, among other variables (Bryant et al., 2011). However, it would be extremely difficult to replicate our protocol in PD patients “off” medication, as walking for 15 min without interruption may be nearly impossible for them. Finally, our experimental procedure may have induced a confounding effect of the instructions (i.e., regular stopping and starting initiated by the experimenter). We minimized this by removing the first and last few strides of each trial before the stitching procedure. Further studies could test participants walking shorter trials and stopping (and starting again) whenever they want to, or on contrary starting and stopping at very predictable points (e.g., walking one lap at a time).

## Conclusion

The present study demonstrates that stitching together short gait trials to create a longer time series is not suitable for the analysis of individual stride-to-stride dynamics. While DFA values were not significantly different between conditions for all three groups, ICC coefficients revealed a very poor between-trial reliability of DFA. This result empirically confirms that investigations about locomotor control through the application of fractal analysis require stride time series to be collected continuously.

## Author Contributions

VM contributed conception and design of the study, participated in data analyses and interpretation of results, did literature searches, and drafted and wrote the manuscript. NR assisted in participant recruitment and data collection, helped to assess participants’ eligibility, participated in data analyses, helped to draft the manuscript, and revised the manuscript. AH assisted in participant recruitment, assessed participant eligibility, participated in the interpretation of results, helped to draft the manuscript, and revised the manuscript. All authors read and approved the final manuscript.

## Conflict of Interest Statement

The authors declare that the research was conducted in the absence of any commercial or financial relationships that could be construed as a potential conflict of interest.

## Abbreviations

CV: coefficient of variation
DFA: detrended fluctuation analysis
HE: healthy elderly
HY: healthy young
ICC: intra-class correlation
PD: Parkinson’s disease
