# Supplementary material for: Gait Dynamics in Parkinson’s Disease: Short Gait Trials “Stitched” Together Provide Different Fractal Fluctuations Compared to Longer Trials
Source: Front Physiol. 2018 Jul 9;9:861. doi: 10.3389/fphys.2018.00861 (PMC6047485; doi:10.3389/fphys.2018.00861)
Supplement: Supplementary file 1 [file Data_Sheet_1.zip › Supplementary Material.docx]

Supplementary Material

**Gait dynamics in Parkinson’s disease: Short gait trials ‘stitched’ together provide different fractal fluctuations compared to longer trials**

Vivien Marmelat^*^, Nicholas R. Reynolds, Amy Hellman

*Correspondence: Vivien Marmelat; vmarmelat@unomaha.edu

# Supplementary Data

**S1 Table. Stride time series**. Matlab matrix composed of 9 cells, each cell containing stride time series of a group in a block. Cells rows correspond to groups (HY, HE and PD, from top to bottom), while cells columns correspond to blocks (’15 min’, ‘3 min’, ’30 sec’, from left to right).

**S2 Table. ICC of DFA values from artificially stitched time series.** Matlab matrix composed of 3 variables: R (ICC values), UB (Upper bound of 95 % confidence interval) and LB (Lower bound of 95 % confidence interval). Each column represents an experimental group: HY, HE and PD, from first to third column.
